# The in vitro free radical scavenging activity of tenidap, a new dual cyclo-oxygenase and 5-1ipoxygenase inhibitor

**DOI:** 10.1155/S0962935192000231

**Published:** 1992

**Authors:** C. S. Lau, J. J. F. Belch

**Affiliations:** University Department of Medicine Ninewells Hospital and Medical School Scotland Dundee DD1 9SY UK

## Abstract

Tenidap, a new anti-inflammatory drug, is presently undergoing clinical studies as a treatment for rheumatoid arthritis (RA). Early pilot work has shown it to be of some benefit. Tenidap is a dual inhibitor of cyclo-oxygenase and 5-lipoxygenase enzymes. It has also been shown to modify white blood cell behaviour such as interleukin-1 production, monocyte differentiation and neutrophil degranulation. As free radicals (FRs) have been implicated in the pathogenesis of RA, we used an in vitro assay system developed by Misra and Fridovich to assess if tenidap has FR scavenging effects. Our study shows, for the first time, that tenidap has general FR scavenging effects although no effect on the superoxide anion (${\text{O}}_{2}^{\cdot -}$) could be demonstrated. This effect occurred in a dose-dependent manner at concentrations above 20 μg/ml (p < 0.005, Mann-Whitney U-test). As the therapeutic range of tenidap in serum is between 15 and 30 μg/ml such FR scavenging activity may be clinically relevant in the treatment of RA. Ex vivo confirmation of this possibility is underway.


Mediators of Inflammation, 1, 141–143 (1992)

### The *in vitro* free radical scavenging activity of tenidap, a new dual cyclo-oxygenase and 5-lipoxygenase inhibitor

C. S. Lau MB ChB MRCP[CA] and
J. J. F. Belch MD FRCP

University Department of Medicine, Ninewells Hospital and Medical School, Dundee DD1 9SY, Scotland, UK

[CA] Corresponding Author

**TENIDAP, a new anti-inflammatory drug, is presently undergoing clinical studies as a treatment for rheumatoid arthritis (RA). Early pilot work has shown it to be of some benefit. Tenidap is a dual inhibitor of cyclo-oxygenase and 5-lipoxygenase enzymes. It has also been shown to modify white blood cell behaviour such as interleukin-1 production, monocyte differentiation and neutrophil degranulation. As free radicals (FRs) have been implicated in the pathogenesis of RA, we used an *in vitro* assay system developed by Misra and Fridovich to assess if tenidap has FR scavenging effects. Our study shows, for the first time, that tenidap has general FR scavenging effects although no effect on the superoxide anion ($O_2^{\bullet-}$) could be demonstrated. This effect occurred in a dose-dependent manner at concentrations above 20 μg/ml ($p < 0.005$, Mann-Whitney U-test). As the therapeutic range of tenidap in serum is between 15 and 30 μg/ml such FR scavenging activity may be clinically relevant in the treatment of RA. *Ex vivo* confirmation of this possibility is underway.**

**Key words:** Free radicals, 5-Lipoxygenase inhibitor, Non-steroidal anti-inflammatory drug, Rheumatoid arthritis

### Introduction

Although the pathophysiology of rheumatoid arthritis (RA) is not fully understood, certain mediators have been shown to be important in the development of inflammation. The role of prostaglandins (PGs), metabolites of arachidonic acid (AA) via the action of cyclo-oxygenase (CO), is well established.[1] Recently, leukotrienes (LTs), 5-lipoxygenase (5-LP) metabolites of AA, have also been shown to be involved in the inflammatory reactions in RA.[2] Additionally, previous studies have shown reactive oxygen free radicals (FRs) may be important in the development of inflammatory synovitis.[3]

Non-steroidal anti-inflammatory drugs (NSAIs) are widely used in RA and other inflammatory arthropathies. They reduce inflammation via their ability to inhibit CO.[1] However, most NSAIs at therapeutic levels do not affect LT synthesis. Dual inhibitors of CO and 5-LP might therefore be expected to be superior to CO inhibitors alone and such agents may offer therapeutic advantages in patients with RA. Tenidap sodium, a novel anti-inflammatory agent, has been shown to have combined CO and 5-LP inhibitory activity[4] and has been reported to be efficacious in short-term studies of patients with RA.[5] Another study has shown tenidap to inhibit *in vitro* activation of human neutrophil collagenase partly due to interference with the production of superoxide radicals ($O_2^{\bullet-}$).[6] The mechanism of inhibition of $O_2^{\bullet-}$production by tenidap is not fully understood although the authors suggested that this may be a secondary phenomenon related to the inhibition of the signal transduction pathway in the neutrophil rather than a direct interaction between tenidap and $O_2^{\bullet-}$.

However, if tenidap has antioxidant properties it may be of further therapeutic advantage in patients with RA. For this reason we used an *in vitro* assay to assess if tenidap was capable of scavenging *in vitro* production of $O_2^{\bullet-}$ and other general FRs.

### Methods

The *in vitro* assay used in this experiment was originally developed by Misra and Fridovich[7] for the estimation of superoxide dismutase (SOD) activity but it may also be used to differentiate between specific $O_2^{\bullet-}$ and general FR scavenging activity. This assay is based on the photo-oxidation of o-dianisidine ($DH_2$) sensitized by riboflavin (Rb) (Fig. 1). Rb absorbs a photon and becomes electronically excited (Rb*). Rb* oxidizes $DH_2$ and this leads to the formation of flavin semiquinone (RbH) and the dianisidine radical ($DH^{\bullet}$). $DH^{\bullet}$ is then involved in further reactions. It may either dismute to yield divalently oxidized dianisidine (D), which absorbs at 460 nm and is measurable by an ultraviolet/visible spectrophotometer, or be reduced by $O_2^{\bullet-}$ ion to form $DH_2$ and oxygen ($O_2$). The $O_2^{\bullet-}$ ion involved in the latter reaction is

© 1992 Rapid Communications of Oxford Ltd

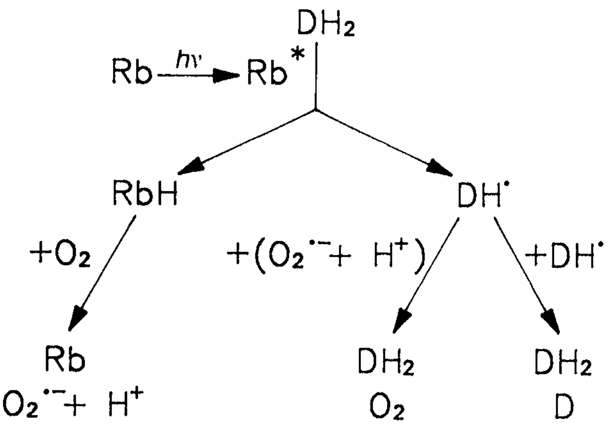

FIG. 1. Photo-oxidation of o-dianisidine. $DH_2$—dianisidine; DH•—dianisidine radical; D—oxidized dianisidine (measurable at 460 nm); Rb—riboflavin; Rb*—excited riboflavin; RbH—flavin semiquinone; $h\nu$—energy of photon of light; $H^+$—hydrogen ion; $O_2$—oxygen; $O_2^{\bullet -}$—superoxide anion.

supplied by the reduction of $O_2$ by RbH. Thus, an agent with $O_2^{\bullet -}$ scavenging activity will divert DH• to the oxidative reaction with enhanced formation of D, thereby increasing the absorbance at 460 nm. On the other hand, a general radical scavenging agent will remove DH•, which reduces the formation of D, thereby decreasing the absorbance at 460 nm.

*Materials:* Tenidap sodium (CP-66,248) (Fig. 2) was obtained from Pfizer Central Research (Sandwich UK). Riboflavin and o-dianisidine were purchased from Sigma Chemicals Ltd. Riboflavin solution ($1.3 \times 10^{-5}$ M) was prepared in 0.01 M potassium phosphate buffer, pH 7.5, and o-dianisidine solution ($10^{-2}$ M) was prepared in ethanol. Tenidap was dissolved in 0.01 M potassium phosphate buffer solution.

Illumination for the photochemical reaction was provided by a pair of parallel 10 watt white fluorescent tubes mounted 15 cm apart in an open-ended box lined with aluminium foil. These tubes provided a constant source of wide-band radiation.

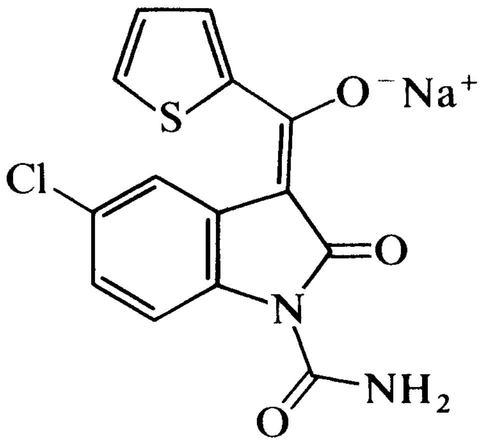

FIG. 2. The molecular structure of tenidap [5-chloro-2,3-dihydro-2-oxo-3-(2-thienylcarbonyl)-indole-1-carboxamide].

*Assay procedure:*

*Control sample.* Sixty μl of $DH_2$ was added to a cuvette containing 2.94 ml of Rb solution. Absorbance of light was measured at 460 nm using a Philips PU 8680 VIS/NIR kinetics spectrophotometer. The cuvette was then placed inside the illumination box for 4 min after which absorbance was measured again at 460 nm. The change in absorbance of this solution was used as the control and was referred to as zero per cent inhibition on the assay.

*Tenidap sample.* Sixty μl of tenidap solution was added to 2.88 ml of Rb solution followed by 60 μl of $DH_2$ solution and the measurements were carried out as above. Each measurement was repeated on six occasions and percentage inhibition on the assay was calculated at each occasion. Final concentrations of tenidap at 5, 10, 15, 20, 22.5, 25, 30, 35, 40, 50 and 60 μg/ml were used.

*Statistical analysis:* The Mann–Whitney U-test was used to analyse differences between data obtained from the control sample and those from the tenidap samples. A $p$-value of $<0.05$ was considered statistically significant.

### Results

Figure 3 shows the mean (SEM) percentage inhibition on the assay by increasing doses of tenidap. Absorption at 460 nm was decreased significantly when the final concentrations of tenidap were above 20 μg/ml. The higher the concentration of tenidap, the greater the inhibition (all $p < 0.005$).

### Discussion

This study shows, for the first time, that tenidap is a general radical scavenger. We show that it has no effect on $O_2^{\bullet -}$ but it has general radical

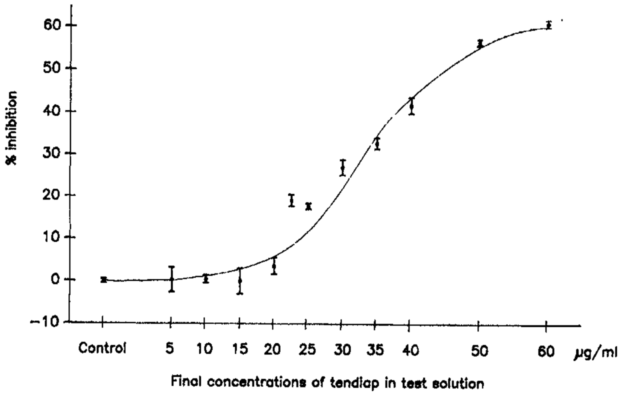

FIG. 3. Percentage inhibition on the assay by tenidap. Results are expressed as mean (SEM).

scavenging effects which are dose-related at final concentrations above 20 μg/ml. This may explain its apparent usefulness in RA, as the therapeutic range of tenidap sodium concentrations in serum is between 15 and 30 μg/ml (Pfizer Central Research).

The use of the *in vitro* assay of Misra and Fridovich to assess FR scavenging properties of other therapeutic agents has been validated previously. For example, the FR scavenging effects of captopril, an angiotensin converting enzyme inhibitor, and gliclazide, a sulphonylurea hypoglycaemic agent, have been confirmed using this assay.[8,9] However, this assay system does not demonstrate which part of the test molecule is responsible for its FR scavenging effect. FRs are chemical species (molecule or atom) with an unpaired electron which renders them reactive. In order to achieve a more stable state, FRs try to accept or donate electrons to other compounds. Both captopril, which possesses a sulphydryl group, and gliclazide, with an azabicyclo-octyl ring, are capable of accepting an electron and undergoing conformational changes, thus stabilizing the reactive FRs. It is possible that the carbonyl or carboxamide structure on the tenidap molecule has similar reducing properties and inhibits the production of DH• by accepting an electron from the excited riboflavin. However, further *in vitro* studies are required to confirm this hypothesis.

Free radicals are thought to play an important role, both directly and indirectly, in the inflammatory process.[10] There is now much evidence supporting a pathological role of oxygen FRs in RA. For example, cells that are present in the inflamed joint, such as macrophages, neutrophils, lymphocytes and endothelial cells are all capable of producing FRs when isolated. FRs have been shown in *in vitro* and animal studies to cause damage to cartilage cells and inhibit a proteoglycan synthesis.[3] In patients with RA, products of oxidative damage are found in serum and synovial fluid and have been shown to correlate with disease activity.[11]

In conclusion, tenidap is a new anti-inflammatory drug which, unlike other ordinary NSAIs, has additional chemical properties including the inhibition of 5-LP[4] and neutrophil release reactions.[5,6] Other authors have also shown it to inhibit the synthesis of interleukin-1.[12] Our study suggests that tenidap has further therapeutic properties with an ability to scavenge FRs which may be of relevance in preventing tissue injury in RA. Such activity is particularly interesting as previous *in vitro* studies have shown 5-LP products, in particular $LTB_4$, to be reactive towards FRs, and it has been suggested that such reactivity may partly explain the chemotactic activity exhibited by LTs.[13] An *ex vivo* study of the effects of tenidap on FR activity in patients with RA is currently underway in a double-blind study. The correlation between tenidap's effects on RA activity, and the production and removal of FRs will be assessed. It is hoped that results from this study will improve our understanding of the pathophysiological role of free radicals in rheumatoid arthritis.

### References

1. Higgs GA, Moncada S, Vane JR. Eicosanoids in Inflammation. *Ann Clin Res* 1984; **16**: 287–299.
2. Belch JJF. Eicosanoids and Rheumatology: Inflammatory and vascular aspects. *Prostaglandins, Leukotrienes and Essential Fatty Acids* 1989; **36**: 219–234.
3. Merry P, Winyard PG, Morris CJ, Grootveld M, Blake DR. Oxygen Free Radical, Inflammation, and Synovitis: The current status. *Ann Rheum Dis* 1989; **48**: 864–870.
4. Moilanen E, Alanko J, Asmawi MZ, Vapaatalo H. CP-66,248, a New Anti-inflammatory Agent, is a Potent Inhibitor of Leukotriene $B_4$ and Prostanoid Synthesis in Human Polymorphonuclear Leucocytes *in vitro*. *Eicosanoids* 1988; **1**: 35–39.
5. Smith DM, Johnson JA, Loeser R, Turner RA. Evaluation of Tenidap (CP-66,248) on Human Neutrophil Arachidonic Acid Metabolism, Chemotactic Potential and Clinical Efficacy in the Treatment of Rheumatoid Arthritis. *Agents & Actions* 1990; **31**: 102–109.
6. Blackburn WD, Loose DL, Heck LW, Chatham WW. Tenidap, in Contrast to Several Available Nonsteroidal Antiinflammatory Drugs, Potently Inhibits the Release of Activated Neutrophil Collagenase. *Arthritis Rheum* 1991; **34(2)**: 211–216.
7. Misra HP, Fridovich I. Superoxide Dismutase: A photochemical augmentation assay. *Arch Biochem Biophys* 1977; **187**: 303–307.
8. Chopra M, Scott N, McMurray J, McLay J, Bridges A, Smith WE, Belch JJF. Captopril: A free radical scavenger. *Br J Clin Pharmacol* 1989: **27**: 396–399.
9. Scott NA, Jennings PE, Brown J, Belch JJF. Gliclazide: A general free radical scavenger. *Eur J Pharmacol* 1991: **208**: 175–177.
10. Dormandy TL. An Approach to Free Radicals. *Lancet* 1983; **ii**: 1010–1013.
11. Rowley DA, Gutteridge JMC, Blake DR, Farr M, Halliwell B. Lipid Peroxidation in Rheumatoid Arthritis: Thiobarbituric acid-reactive material and catalytic iron salt in synovial fluid from rheumatoid patients. *Clin Sci* 1984; **66**: 691–695.
12. MacDonald B, Loose L, Rosenwasser LJ. The Influence of a Novel Arachidonate Inhibitor, CP-66,248, on the Production and Activity of Human Monocyte IL-1. *Arthritis Rheum* 1988; **31(4, Suppl)**: S17.
13. Chopra M, Belch JJF, Smith WE. A Comparison of the Free Radical Scavenging Activity of Leukotrienes and Prostaglandins. *Free Rad Res Comms* 1988; **5(2)**: 95–99.

ACKNOWLEDGEMENTS. We are grateful to Pfizer Central Research (Sandwich UK) for financial support and for tenidap sodium. JB is supported by the Sir John Fisher Foundation.

**Received 18 October 1991;
accepted in revised form 6 February 1992**
